# Screening for symptoms of childhood traumatic stress in the primary care pediatric clinic

**DOI:** 10.1186/s12887-024-04669-3

**Published:** 2024-03-27

**Authors:** Kristine A. Campbell, Kara A. Byrne, Brian L. Thorn, Lindsay Shepard Abdulahad, R. Neal Davis, Lisa L. Giles, Brooks R. Keeshin

**Affiliations:** 1 Department of Pediatrics, University of Utah, Primary Children’s Hospital Eccles Outpatient Building, 81 North Mario Capecchi Drive, Salt Lake City, UT 84113 USA; 2 University of Utah Kem C. Gardner Policy Institute David Eccles School of Business, 411 East South Temple Street, Salt Lake City, UT 84111 USA; 3 Intermountain Healthcare Hillcrest Pediatrics, 5063 S. Cottonwood St, Ste 160, Murray, UT 84157 USA; 4 Salt Lake City, UT, USA; 5 Department of Public Health and Caring Science, Child Health and Parenting (CHAP), Uppsala University, Uppsala, Sweden

**Keywords:** Traumatic stress, Screening, Mental health, Primary care

## Abstract

**Background:**

Childhood traumatic experiences may result in post-traumatic stress disorder. Although pediatricians are encouraged to address these traumas in clinical encounters, measures of childhood traumatic stress have not been adopted by primary care clinicians. In this study, we describe the feasibility and potential utility of the UCLA Brief Screen, a validated screener for childhood traumatic stress symptoms, in pediatric primary care clinics.

**Methods:**

Children 6–17 years of age presenting for routine well-child care in community-based pediatric clinics were eligible for traumatic stress screening. We described the feasibility and acceptability of screening based on screener adoption by eligible pediatric clinicians. We assessed the potential utility of screening based on prevalence and distribution of potentially traumatic events and traumatic stress symptoms in this general pediatric population. Finally, we compared results of the UCLA Brief Screen with those of the Patient Health Questionnaire-A to evaluate associations between symptoms of traumatic stress, depression, and suicidality among adolescents in this community setting.

**Results:**

14/18 (77.8%) pediatric clinicians in two clinics offered an adapted UCLA Brief Screen during 2359/4959 (47.6%) eligible well-child checks over 14 months. 1472/2359 (62.4%) of offered screeners were completed, returned, and scored. One-third (32.5%) of completed screeners captured a potentially traumatic event experience described by either children or caregivers. Moderate to severe traumatic stress symptoms were identified in 10.7% and 5.2% of patients, respectively. Concurrent depression screening revealed that 68.3% of adolescents with depressive symptoms reported a potentially traumatic event (PTE) and 80.5% had concurrent traumatic stress symptoms. Adolescents reporting a PTE were 3.5 times more likely to report thoughts of suicide or self-harm than those without this history.

**Conclusions:**

Results from this pilot study suggest that traumatic stress screening in the pediatric primary care setting may be feasible and may identify and classify mental health symptoms missed with current screening practices for depression. The prevalence of PTEs and traumatic stress symptoms associated with PTEs support the potential utility of a standardized screening in early identification of and response to children with clinically important symptoms of childhood traumatic stress. Future research should evaluate meaningful clinical outcomes associated with traumatic stress screening.

## Background

Potentially traumatic events (PTEs), including serious injury, interpersonal violence, natural disasters and loss, are common experiences for youth in the United States. One-third of U.S. children experience a PTE by 11 years of age, 60–80% of young adults report at least one PTE during childhood, and 20–48% report multiple PTEs [1–3]. Childhood PTEs are associated with diminished physical and mental health, as well as increased lifetime risks for post-traumatic stress disorder (PTSD), suicide, and self-harm [4–7]. Effective, evidence-based therapies for traumatic stress are well-established and increasingly available to children with symptoms emerging after a PTE [8–10]. Unfortunately, traumatic stress can be easily mistaken for childhood depression or attention deficit disorder in the absence of screening to identify PTEs and traumatic stress symptoms [11, 12]. If PTEs and traumatic stress are not correctly identified by health care clinicians, children may experience unnecessary delays in accessing effective trauma care.

Recognition of the lifelong health risks associated with childhood trauma and the availability of effective interventions for traumatic stress has led professional organizations to call for screening for these exposures in primary health care settings [13, 14]. The American Academy of Child and Adolescent Psychiatry (AACAP) recommends routine screening for PTEs and for symptoms of traumatic stress and the American Academy of Pediatrics (AAP) recommends “active screening for precipitants of toxic stress” in the primary care pediatric setting [14, 15]. These recommendations have spread rapidly across popular press and social media, contributing to lay expectations that primary care clinicians understand and respond to PTEs and other adverse childhood experiences (ACEs) within the clinical setting [16].

The practice of a checklist screening for exposure to a defined set of potentially traumatic childhood experiences has important limitations. There is no single checklist that captures every experience that places children at risk for traumatic stress. A count of ACEs or PTEs may overestimate the risk of traumatic stress in those children who are thriving in spite of adversities or accumulation of traumatic experiences. At the same time, an ACE score will fail to identify an urgent need for evidence-based intervention among those children struggling with clinically significant traumatic stress symptoms after just one or two exposures. As an example, the popular 10-item ACEs screener, identifies the child with a distant history of maltreatment but fails to capture the child with a recent episode of life-threatening anaphylaxis or the teen with a parent facing the possibility of deportation [4, 17, 18]. More importantly, while ACEs scores can be critical to understanding population-level health disparities, it does not provide actionable clinical decision-making support in the context of an individual patient encounter [19–22].

This emphasis on ACEs screening may have overshadowed important efforts to improve recognition of the effects of PTEs on patients in the pediatric clinic. Primary care screening for the presence and severity of symptoms of traumatic stress may be more useful for clinical decision-making than a simple accounting of selected PTEs. In comparison to screening for autism and adolescent depression, however, screening for traumatic stress in the primary care setting is rare. Several barriers to screening for traumatic stress may exist. First, many measures of symptoms of childhood traumatic stress are comprehensive diagnostic instruments that are not practical for widespread use in a primary care clinical setting [23]. The UCLA Brief Screen for Trauma (UCLA Brief Screen) was recently validated in a clinical sample of children at high risk for PTSD receiving mental health services following a known PTE exposure(s) [24]. The 11-item UCLA Brief Screen identifies traumatic stress symptoms predictive of PTSD, but has not been implemented in a primary care setting. Second, guidance in the response to childhood traumatic stress in the primary care settings has been limited. Recent attention to the role of the primary care pediatrician in care of the child who has experienced a PTE provides an opportunity to improve the recognition of and response to childhood traumatic stress in this accessible clinical setting [12, 13, 25, 26].

The current project describes the introduction of the UCLA Brief Screen as the first step in a comprehensive care process model developed to support recognition of and response to childhood traumatic stress in the pediatric clinic. In this pilot study, we examined adoption of the UCLA Brief Screen piloted by clinicians in two primary care practice settings. First, we tested whether the report of any patient or parent-identified PTE, defined as a “violent or very scary or upsetting” experience, could be used in place of a narrow checklist of PTEs or ACEs. We hypothesized that a positive response to this simple screening question would be associated with symptoms of traumatic stress, supporting an efficient, stepped screening approach in a busy primary care setting. Next, we described the distribution of traumatic stress symptoms reported in the routine health screenings gathered during annual well child care checks. Finally, we tested the hypotheses that traumatic stress symptoms are distinct from symptoms of depression already captured by common pediatric screening and that traumatic stress symptoms are independently associated with thoughts of suicide and/or self-harm.

## Methods

### Study design and procedures

The adapted UCLA Brief Screen was piloted in two general pediatric clinics from 5/1/18 through 6/30/19. The study team provided participating clinicians with a 45-minute training on use of the UCLA Brief Screen, identification of traumatic stress symptoms, and recommendations for in-office interventions and referrals. While our team remained available for technical assistance and consultation, no further training was conducted in the course of this pilot.

We evaluated screener adoption in day-to-day practice of eligible pediatric clinicians. We then described the cross-sectional association between a simple report of a “violent or very scary or upsetting” experience (“PTE exposure”) and traumatic stress symptoms, and the association between traumatic stress symptoms and symptoms of depression and suicidality among children 6–17 years of age seen for routine well child care in these clinics. All study procedures were deemed exempt by the University of Utah IRB (IORG0000072).

### Setting

This pilot was conducted in two pediatric clinics served by 18 clinicians located in a single state in the United States Intermountain West. A pediatric clinician at one clinic served as a project champion and facilitated introduction of the screener to both clinics (author RND).

### Participants

Primary care participants were children 6–17 years of age seen for an annual well child check by a trained provider during the 14-month study timeframe. Only the first eligible encounter was used for children with two or more well child checks over this period. Children with missing demographics, social risk indicators, and/or diagnostic data were excluded.

### Data collection

All screening in the current study was collected on paper as part of routine clinical practice during annual well-child checks for children 6–17 years of age. Parents completed screening instruments including the adapted UCLA Brief Screen for children 6–10 years of age. Adolescents 11–17 years of age completed self-report screening instruments including the adapted UCLA Brief Screen and the Patient Health Questionnaire (PHQ-A). Clinicians reviewed completed screeners before or during the clinic visit.

All screeners offered were collected by the study team and entered into REDCap, a secure web platform for managing research databases hosted by the University of Utah. Screener results were linked to the electronic medical record to obtain individual demographics and health history.

### Measures

#### Childhood traumatic stress

The UCLA Brief Screen is an 11-item measure derived from the UCLA PTSD Reaction Index as a valid rapid screener for childhood traumatic stress symptoms [24, 27]. Studies of the UCLA Brief Screen found excellent internal consistency (α = 0.90) in a diverse sample of children age 7–18 years receiving outpatient mental health treatment for a known PTE. Receiver operating characteristics (ROC) analyses (comparing the UCLA Brief Screen to the Clinician Administered PTSD Scale for DSM-5- Child/Adolescent Version (CAPS-CA-5) among children with known PTE) demonstrated support for the measure’s clinical utility in discrimination between children with and without PTSD based on a cut-off score of ≥ 21.

In the current study, we wanted to understand the feasibility of screening generally healthy children for symptoms of childhood traumatic stress during a routine well child visit with a primary care clinician. While questions to elicit traumatic stress symptoms are unchanged from the validated UCLA Brief Screen, we made four pragmatic adaptations to fit the needs of clinicians, parents and patients in this distinct setting (Table 1). These included (1) an option for parent completion of the screener for younger children, (2) a simple capture of a PTE exposure as a “violent or very scary or upsetting event” rather than a more structured interview, (3) a tiered scoring system to support earlier identification of and response to emerging traumatic stress symptoms, and (4) the addition of a suicide screening question to assure recognition of and response to those children at highest risk for self-harm.

**Table 1:** Pragmatic adaptions to the UCLA brief screen for the pediatric traumatic stress screening tool

Validated UCLA Brief Screen	Adapted UCLA Brief Screen	Rationale
Self-report form	Parent and self-report forms	The UCLA Brief Screen relied on patient self-report of trauma and trauma symptoms in trauma-exposed youth 7–18 years of age seen for mental health concerns in a specialized clinical setting. In general pediatric clinics, adolescents are often asked to self-report symptoms of depression or anxiety at 11 years of age. Due to concerns regarding literacy and comfort related to self-report of PTEs among younger patients in a general pediatric setting, we developed a parent-report UCLA Brief Screen version for children 6–10 years of age.
Detailed history of trauma exposure	Brief capture of any potentially traumatic event (PTE)	The UCLA Brief Screen was validated among youth with a trauma history captured with a detailed Trauma History Profile of the full-scale UCLA PTSD Reaction Index. The Profile lists 14 specific PTEs, and a final option to endorse any other “really scary or upsetting” experience. We adapted this final question to capture parent or child endorsement of either a recent or past “violent or very scary or upsetting event” as an indicator of PTE exposure and examined whether this history was positively associated with trauma symptoms reported on the UCLA Brief Screen among general pediatric patients.
Trauma symptom severity based on PTSD probability	Trauma symptom severity based on need for intervention	The UCLA Brief Screen used multilevel diagnostic likelihood ratios (DLRs) to identify scores associated with low (0–20), moderate (21–35), and high (36–44) risk for PTSD. In a primary care setting, children with milder symptoms of traumatic stress following a PTE may benefit from early identification, assessment, and intervention even in the absence of PTSD based on formal diagnostic criteria. Informed by cut points described in the initial validation study, we adapted the scoring system to classify children as none/mild (0–10), moderate (11–20) or severe (21–44) symptoms of traumatic stress.
Trauma screening only	Trauma + suicide screening	We added a screening question for risk of suicide and/or self-harm (Question 9 from the PHQ-A). Positive responses prompted full screening with the Columbia Suicide Severity Rating Scale.

#### Depressive symptoms

In the participating clinics, adolescent patients are typically screened for depressive symptoms with the adolescent version of the Patient Health Questionnaire (PHQ-A) during routine well child care beginning at 11 years of age. The PHQ-A includes a scoreable 9-item self-report measure that assesses severity of depressive symptoms in adolescents and is commonly used in general pediatric practices. The total score ranges from 0 to 27, with higher scores indicating higher depressive symptoms. A total score of ≥ 11 is recommended as an optimal cut point for concern for adolescent depression in the primary care setting [28–30].

#### Suicide and/or self-harm

Risk for suicide and/or self-harm was established based on a positive response to question 9 on the PHQ-A (“Over the past 2 weeks, how often have you (your child) been bothered by thoughts that you (he/she) would be better off dead or of hurting yourself (him or herself) in some way?”). Clinicians noting any response other than “not at all” to this question were guided towards additional evidence-based screening and response tools [31].

#### Child demographics and health history

Child age, sex, race, diagnoses, and social risks were obtained from the electronic medical record, providing patient-level aggregate variables to reflect pediatric medical complexity and mental health concerns [32, 33]. Social risk was represented with an Area Deprivation Index (ADI) reflecting a census block-group indicator of relative deprivation based on 17 U.S. Census measures of social determinants of health [34].

#### Potentially traumatic events (PTE)

A PTE was identified if a parent or child endorsed a recent or past “violent or very scary or upsetting” experience on the screener. A free-text descriptor of this experience was elicited to support a relevant clinical response by the provider.

### Analysis

To understand the feasibility of screening for traumatic stress in a primary care setting, we described adoption of the UCLA Brief Screen. Adoption was based on the proportion of eligible clinicians administering the screener and the proportion of eligible patients receiving and completing the screener. Completed screeners were defined as (1) any response to PTE exposure questions and (2) at least one symptom question response. We described potential bias in screener implementation with bivariate comparison of eligible children with and without screener administration or completion.

To understand the potential utility of the UCLA Brief Screen in a primary care setting, we described prevalence of reported PTE exposures and distribution of traumatic stress symptoms among children with completed screeners. To understand the usefulness of a single, open-ended question related to PTE exposure rather than a PTE questionnaire, we examined bivariate associations between a report of a recent or past “violent or very scary or upsetting” experience and traumatic stress symptom severity. Adjusting for potential confounders, we described the relative risk (ARR) for traumatic stress based on a report of at least one PTE. Finally, we examined the overlap between symptoms of traumatic stress, depression, and suicidality with bivariate comparisons of results of the UCLA Brief Screen, the PHQ-A score, and question 9 of the PHQ-A among dual-screened adolescents. All analyses were performed with STATA SE 15.1 (College Station, TX).

## Results

### Feasibility of screening for childhood traumatic stress

Over the course of one year, 14 out of 18 (77.8%) pediatric clinicians adopted the adapted UCLA Brief Screen (6/6 Clinic A providers and 8/12 Clinic B providers). We included 4959 of 5130 (96.7%) children with complete demographic, social risk indicator, and diagnostic data who were seen by participating clinicians during this time period (Table 2). Across clinics, 2359/4959 (47.6%) children 6–17 years of age were offered a screener during a well child check. This implementation varied widely by individual participating clinicians, with anywhere from 2.2 to 82.1% of eligible patients offered a screener across different clinicians. Overall, this improved significantly over time, beginning with 40.5% of patients offered screeners in the first quarter of the pilot to 57.2% in the final quarter (*p* < 0.001). Of 2359 screeners offered during eligible visits, 1472 (62.4%) were completed. Average completion rates improved over the study timeframe from 51.0 to 65.5% but varied widely across individual clinicians (29.3–93.8%). Screener introduction was more common among adolescent patients, patients living in higher social risk communities, and patients of Hispanic/Latinx ethnicity. Screener completion was more common among adolescent patients and patients living in lower social risk communities (Table 2).

**Table 2:** Children 6–17 years old eligible for childhood traumatic stress screening

	Eligible for Screening *n* = 4959 (%)	Offered Screening *n* = 2359 (%)	Completed Screening *n* = 1472 (%)	Reported PTE on Screener *n* = 478 (%)
Age*^				
6–10 years (parent-report)	2619 (52.8)	1149 (48.7)	661 (44.9)	205 (42.9)
11–17 years (self-report)	2340 (47.2)	1210 (51.3)	811 (55.1)	273 (57.1)
**Sex**				
Female	2584 (52.1)	1229 (52.1)	783 (53.2)	268 (56.1)
Male	2375 (47.9)	1130 (47.9)	689 (46.8)	210 (43.9)
**Race**				
Majority/White	4482 (90.4)	2135 (90.5)	1336 (90.8)	436 (91.2)
Minority/Non-White	477 (9.6)	224 (9.5)	136 (9.2)	42 (8.8)
Ethnicity*				
Non-Hispanic/Latinx	4242 (85.5)	1991 (84.4)	1258 (85.5)	405 (84.7)
Hispanic/Latinx	717 (14.5)	368 (15.6)	214 (14.5)	73 (15.3)
Area deprivation index (ADI)*^† ^**1**^				
Low social risk (ADI 1–3)	4083 (82.3)	1895 (80.3)	1221 (83.0)	380 (79.5)
High social risk (ADI 4–5)	876 (17.7)	464 (19.7)	251 (17.1)	98 (20.5)
**Mental health history†** ^**2**^				
None	3775 (76.1)	1768 (75.0)	1092 (74.2)	302 (63.2)
Mental health diagnosis	1184 (23.9)	591 (25.0)	380 (25.8)	176 (36.8)
**Medical complexity (PCMA)†** ^**3**^				
None	3193 (64.4)	1504 (63.8)	916 (62.2)	243 (50.8)
Non-complex chronic condition	1172 (23.6)	571 (24.2)	364 (24.7)	135 (28.2)
Complex chronic condition	594 (12.0)	284 (12.0)	192 (13.0)	100 (20.9)

Shading indicates a significant difference between the adjacent columns

* Significant difference between children eligible for and offered UCLA Brief Screen at well-child visit (*p* < 0.05)

^ Significant difference between children offered and completing UCLA Brief Screen at well-child visit (*p* < 0.05)

† Significant difference between children with and without report of PTE on completed UCLA Brief Screen (*p* < 0.05)

1. The Area Deprivation Index (ADI) reflects a census block-group indicator of relative deprivation based on 17 U.S. Census measures of social determinants of health.^31^

2. Mental health history based on AHRQ Clinical Classification Software and adapted using previously described approaches.^30^

3. The Pediatric Medical Complexity Algorithm (PMCA) was developed and tested in children 0 to 18 years old insured by Washington State Medicaid (WA-Medicaid) and can be applied to large datasets representing hospital and health plan utilization by children.^2^

### Potential utility of screening for childhood traumatic stress

#### Potentially traumatic events

**Fig. 1:**
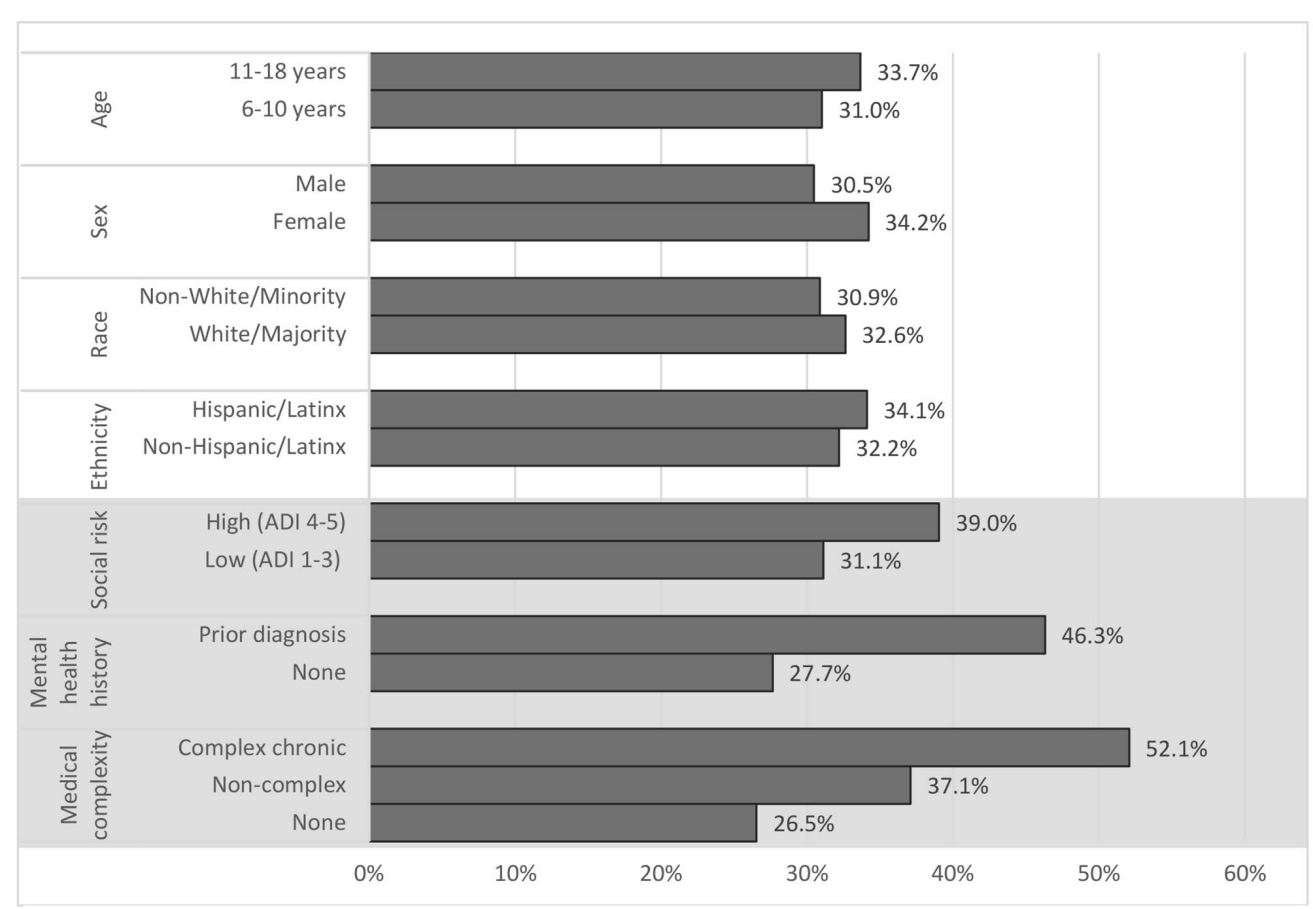
Prevalence of potentially traumatic events reported by caregivers (age 6–10 years) or patients (age 11–17 years) at routine well-child visits *Shaded region reflects increased prevalence of reported PTE associated with high social risk, past mental health diagnosis, and medical complexity (all *p*<0.05)

One in three completed screeners (478/1472, 32.5%) captured a lifetime history of at least one PTE (Table 2). We observed a significantly higher prevalence of PTEs among children with chronic medical conditions compared to those with no chronic medical conditions, children with at least one mental health diagnosis in the past 2 years compared to those without this history, and children living in communities with higher levels of social deprivation compared to children in communities with more social resources (Fig. 1).

#### Traumatic stress symptoms and potentially traumatic events

Symptoms of moderate and severe traumatic stress were reported in 157 (10.7%) and 77 (5.2%) of 1472 patients with completed screeners, respectively. Moderate-to-severe traumatic stress symptoms were reported in 186/478 (38.9%) patients with a history of PTE compared to 48/994 (4.8%) of those without a history of PTE (*p* < 0.001), giving children with a history of any PTE an 8.1 (95% CI 6.0-10.9) times higher unadjusted relative risk of traumatic stress symptoms than those without this history. The sensitivity and specificity of a parent or self-reported PTE for moderate-to-severe traumatic stress were 79.5% (95% CI 77.4%-81.6%) and 76.4% (95% CI 74.2%-78.6%), respectively resulting in a positive predictive value of 38.9% and negative predictive value of 95.2%. In a fully adjusted model, a reported PTE was the only consistent predictor for both moderate-to-severe traumatic stress symptoms (score ≥ 11) identified on the screener. With the more specific outcome of severe traumatic stress symptoms (score ≥ 21), there was increased risk for adolescents, girls, children with a previous mental health diagnosis, and children with non-complex chronic health conditions (Table 3).

**Table 3:** Adjusted relative risk for none/mild, moderate, and severe traumatic stress symptoms on the adapted UCLA brief screen

	None/Mild Traumatic Stress (0–10) *n* = 1238 (84.1%)		Moderate Traumatic Stress (11–20) *n* = 157 (10.7%)		Severe Traumatic Stress (≥ 21) *n* = 77 (5.2%)	
	Prevalence n (%)	Adjusted Risk Ratio ARR (95% CI)	Prevalence n (%)	Adjusted Risk Ratio ARR (95% CI)	Prevalence n (%)	Adjusted Risk Ratio ARR (95% CI)
Potentially Traumatic Event						
none reported	**946 (95.2)**	**(ref)**	**36 (3.6)**	**(ref)**	**12 (1.2)**	**(ref)**
≥ 1 PTE reported	**292 (61.1)**	**0.7 (0.6–0.7)**	**121 (25.3)**	**6.2 (4.3–8.9)**	**65 (13.6)**	**8.4 (4.6–15.3)**
Child age						
6–10 years	588 (89.0)	(ref)	58 (8.8)	(ref)	**15 (2.3)**	**(ref)**
11–17 years	650 (80.2)	1.0 (0.9-1.0)	99 (12.2)	1.4 (1.0, 1.9)	**62 (7.6)**	**2.9 (1.5, 5.6)**
Child sex						
Male	601 (87.2)	(ref)	67 (9.7)	(ref)	**21 (3.1)**	**(ref)**
Female	637 (81.4)	0.9 (0.9-1.0)	90 (11.5)	1.2 (0.8, 1.8)	**56 (7.2)**	**2.4 (1.5, 4.0)**
Ethnicity						
Non-Hispanic	1062 (84.4)	(ref)	129 (10.3)	(ref)	67 (5.3)	(ref)
Hispanic/Latinx	176 (82.2)	1.0 (0.9-1.0)	28 (13.1)	1.2 (0.8–1.8)	10 (4.7)	1.0 (0.5–1.8)
Race						
Majority/White	1113 (83.3)	(ref)	148 (11.1)	(ref)	75 (5.6)	(ref)
Minority/Non-White	125 (91.9)	1.1 (1.0-1.1)	9 (6.6)	0.7 (0.4–1.3)	2 (1.5)	0.4 (0.1–1.5)
Area deprivation index^1^						
Low social risk (ADI 1–3)	1036 (84.9)	(ref)	122 (10.0)	(ref)	63 (5.2)	(ref)
High social risk (ADI 4–5)	202 (80.5)	0.9 (0.9-1.0)	35 (13.9)	1.2 (0.9–1.7)	14 (5.6)	1.1 (0.6–1.8)
Mental health history^2^						
None	967 (88.6)	(ref)	95 (8.7)	(ref)	**30 (2.8)**	**(ref)**
Mental health diagnosis	271 (71.3)	0.9 (0.9-1.0)	62 (16.3)	1.1 (0.8–1.6)	**47 (12.4)**	**2.2 (1.4–3.6)**
Medical complexity^3^						
None	821 (89.6)	(ref)	72 (7.9)	(ref)	**23 (2.5)**	**(ref)**
Non-complex chronic condition	284 (78.0)	0.9 (0.9-1.0)	46 (12.6)	1.2 (0.9–1.7)	**34 (9.3)**	**1.8 (1.1-3.0)**
Complex chronic condition	133 (69.3)	0.9 (0.9-1.0)	39 (20.3)	1.5 (1.0-2.2)	20 (10.4)	1.3 (0.7–2.3)

*Bolded values reflect statistically significant risk of traumatic stress symptoms

1. The Area Deprivation Index (ADI) reflects a census block-group indicator of relative deprivation based on 17 U.S. Census measures of social determinants of health.^31^

2. Mental health history based on AHRQ Clinical Classification Software and adapted using previously described approach

#### Traumatic stress and adolescent depression

**Fig. 2:**
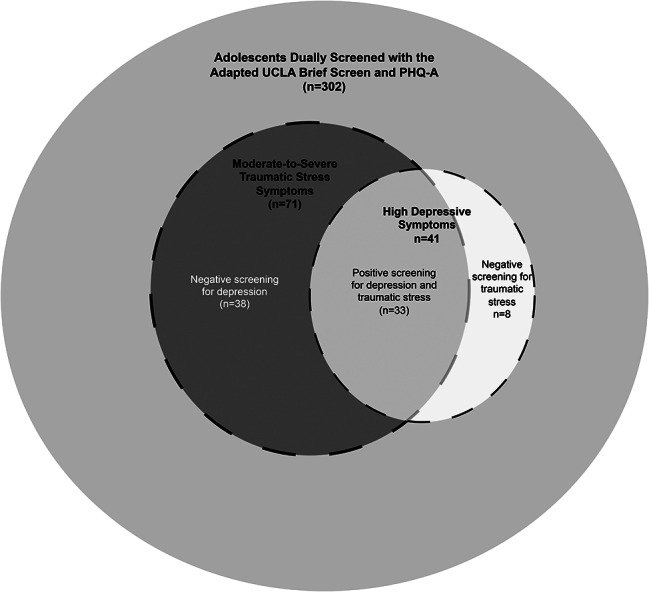
Distribution of depressive and traumatic stress symptoms among dual-screened adolescents in routine well-child visits

302 adolescents screened with the adapted UCLA Brief Screen were simultaneously screened for depressive symptoms with the PHQ-A based on routine clinical practice (Fig. 2). Overall, 71 (23.5%) reported moderate-to-severe symptoms of traumatic stress and 41 (13.6%) had clinically significant symptoms of depression. Examining those who reported significant depression symptoms, two-thirds (28/41, 68.3%) had a history of PTE and only a minority (8/41, 19.5%) had symptoms of depression without evidence of traumatic stress on the UCLA Brief Screen. Of those who reported moderate-to-severe symptoms of traumatic stress, 38/71, 53.5%) had no evidence for depression on the PHQ-A (*p* < 0.001). When limited to 117 dual-screened adolescents reporting a history of PTE, 2/28 (7.1%) had evidence of depression without symptoms of traumatic stress, while 31/57 (54.4%) had moderate-to-severe symptoms of traumatic stress without symptoms of depression (*p* < 0.001).

#### Traumatic stress and adolescent suicidality

Thoughts of suicide or self-harm were endorsed by 29/302 (9.6%) dual-screened adolescents. Adolescents reporting PTE exposure were 3.5 times more likely to report thoughts of suicide or self-harm (20/117, 17.1%) compared to those without this PTE history (9/185, 4.9%) (*p* < 0.001). For those with thoughts of suicide or self-harm, 3 (10.3%) had depressive symptoms only, 7 (24.1%) had traumatic stress symptoms only, and 14 (48.3%) had both depressive and traumatic stress symptoms (*p* = 0.2).

## Discussion

Results of this study suggest that use of a brief screening tool for childhood traumatic stress symptoms adapted for well-child checks in children 6–17 years of age may be feasible within a busy pediatric practice and potentially useful in identifying and responding to patients reporting a PTE. We observed that adoption of the adapted UCLA Brief Screen was successful, with 78% of clinicians implementing the screening during the study timeframe. Overall, 48% of eligible 6–17 year old patients were offered the screener during well-child visits. Use of the screener rose significantly over time without intervention by the study team, suggesting that comfort and acceptance of the tool rose among adopting clinicians during the study timeframe. Almost two-thirds of screeners offered during patient encounters were completed, returned and scored even in the absence of incentives or significant ongoing support of the program. While imperfect, these rates mirror the results of other well-established screening practices in these clinics, with a PHQ-A completion rate of 40% among adolescents in our sample. These findings are not unique, and are comparable to established screening practices for pediatric developmental delay and autism across the U.S [35].

Trauma exposure is a foundational criterion for the identification of traumatic stress, and original validation of the UCLA Brief Screen was conducted in clinical populations with known PTEs [24, 27]. This represented a gap in our understanding of best practices for implementation of the UCLA Brief Screen in a general pediatric population, where comfort with identifying an experience as potentially traumatic is not known. In our study, one-third of patients reported lifetime exposure to at least one PTE based on the self-report of an event experienced as “violent or very scary or upsetting.” This report was associated with an 8-fold increased risk of traumatic stress symptoms, while the absence of a PTE report had a 95.2% negative predictive value for traumatic stress symptoms. Our findings suggest that use of a comprehensive list of possible PTE exposures can be replaced with a single, simple prompt in this primary care setting to determine which patients may benefit from traumatic stress symptom screening.

Given that a “violent or very scary or upsetting” PTE is reported by just one-third of patients, the described stepped approach implemented in this study could significantly reduce “screening fatigue” for both clinicians and patients in the primary care setting while appropriately identifying patients at highest risk for traumatic stress. In this community setting, clinically significant symptoms were identified in 38.9% of children with reported PTE exposure, a positive predictive value similar to or higher than that for well-accepted screeners such as the PHQ-9 [36]. Given this, we believe that any burden of symptom screening based on a PTE self-report is outweighed by the relative ease of this process, the risks and clinical implications associated with traumatic stress, and the potential for psychoeducation for children and parents in this process. Further research is required to determine whether this stepped screening approach is appropriate in very high risk or specialized clinical settings, where the prevalence of PTEs may be higher and the predictive value of a negative PTE report may be lower.

It is important to place the potential benefits of traumatic stress screening in the context of current pediatric practice. The adapted UCLA Brief Screen provides an actionable severity rating of traumatic stress symptoms in children with PTE exposure. The screener also identifies unmet needs among children with special health care needs. Almost half of screened patients with chronic medical conditions—children most likely to be known to a pediatric medical home—had a reported history of PTE. Children with non-complex chronic conditions had 1.8 times higher risk for high levels of traumatic stress symptoms. Finally, our results indicate that dual screening for depression and traumatic stress improves our ability to detect and interpret adolescent depression and suicidality. Routine screening for adolescent depression would have failed to identify over half of all children with clinically important symptoms of traumatic stress in our study. A substantial majority of adolescents with depressive symptoms report a PTE (68%) as well as concurrent traumatic stress symptoms (80%). This is important as symptoms of traumatic stress were common among adolescents with thoughts of suicide and/or self-harm in our clinic population. Screening for and responding to depressive symptoms and suicidality/self-harm without consideration of traumatic stress may lead to unnecessary delays in referral to effective, evidence-based, trauma focused treatments.

These findings must be considered in light of the limitations of our study. We recognize that results observed in these two clinics may not be generalizable to all settings. As a pragmatic pilot project, implementation and adoption relied on the commitment of individual clinics and clinicians. Just half of the eligible clinic patients were offered screeners, likely due to limited uptake by some clinicians as well as day-to-day realities of workflow in a busy clinic. Small but significant differences in who was offered a screener, and in who completed the screener, should be monitored in future studies. Implementation and adoption could be improved with a structured clinical trial of the screening process. Clinics were provided with little feedback on completion rates of trauma screeners, and assumptions related to incomplete screeners in the current analysis may lead to over- or under-estimates of the prevalence of PTEs or traumatic stress in the primary care setting. Practical adaptations to the UCLA Brief Screen made for the primary care setting deserve validation to better understand the sensitivity, specificity, and positive predictive value of screener results and PTSD diagnosis in this setting. Finally, we recognize that justification for screening for traumatic stress in a busy primary care setting deserves thorough evaluation of screening outcomes, including resolution of symptoms based on in-office interventions, engagement with traditional mental health services, or referral to evidence-based trauma therapy for children.

## Conclusions

One-third of children age 6–17 years presenting for routine well child care endorse a history of a “violent, very scary, or upsetting” experience. Almost 40% of these children report moderate-to-severe traumatic stress symptoms that may be traced to these PTEs. Use of the adapted UCLA Brief Screen during routine well child care was feasible in a pediatric primary care setting. Future studies will determine whether this process can support evidence-based decision-making for primary care clinicians working with children with a history of potentially traumatic experiences.

## Data Availability

The datasets used and/or analysed during the current study are available from the corresponding author on reasonable request with appropriate data sharing agreements through the University of Utah.

## Abbreviations

PTE: Potentially Traumatic Event
PTSD: Post Traumatic Stress Disorder
ACE: Adverse Childhood Experience
